# Animal models for pelvic organ prolapse: systematic review

**DOI:** 10.1007/s00192-020-04638-1

**Published:** 2021-01-23

**Authors:** Marina Gabriela M. C. Mori da Cunha, Katerina Mackova, Lucie Hajkova Hympanova, Maria Augusta T. Bortolini, Jan Deprest

**Affiliations:** 1 Centre for Surgical Technologies, Group Biomedical Sciences, KU Leuven, Leuven, Belgium; 2 Department of Development and Regeneration, Woman and Child, Group Biomedical Sciences, KU Leuven, Leuven, Belgium; 3 Institute for the Care of Mother and Child, Third Faculty of Medicine, Charles University, Prague, Czech Republic; 4 Department of Gynecology, Sector of Urogynecology, Universidade Federal de São Paulo, São Paulo, SP Brazil; 5 Pelvic Floor Unit, University Hospitals, KU Leuven, Leuven, Belgium; 6 Department of Development and Regeneration, Center of Surgical Technologies UZ Herestraat, Herestraat49, B3000 Leuven, Belgium

**Keywords:** Ovariectomy, Pregnancy, Parity, Pathophysiology, Age, Menopause

## Abstract

**Introduction and hypothesis:**

We aimed to summarize the knowledge on the pathogenesis of pelvic organ prolapse (POP) generated in animal models.

**Methods:**

We searched MEDLINE, Embase, Cochrane and the Web of Science to establish what animal models are used in the study of suggested risk factors for the development of POP, including pregnancy, labor, delivery, parity, aging and menopause. Lack of methodologic uniformity precluded meta-analysis; hence, results are presented as a narrative review.

**Results:**

A total of 7426 studies were identified, of which 51 were included in the analysis. Pregnancy has a measurable and consistent effect across species. In rats, simulated vaginal delivery induces structural changes in the pelvic floor, without complete recovery of the vaginal muscular layer and its microvasculature, though it does not induce POP. In sheep, first vaginal delivery has a measurable effect on vaginal compliance; measured effects of additional deliveries are inconsistent. Squirrel monkeys can develop POP. Denervation of their levator ani muscle facilitates this process in animals that delivered vaginally. The models used do not develop spontaneous menopause, so it is induced by ovariectomy. Effects of menopause depend on the age at ovariectomy and the interval to measurement. In several species menopause is associated with an increase in collagen content in the longer term. In rodents there were no measurable effects of age apart of elastin changes. We found no usable data for other species.

**Conclusion:**

In several species there are measurable effects of pregnancy, delivery and iatrogenic menopause. Squirrel monkeys can develop spontaneous prolapse.

**Supplementary Information:**

The online version contains supplementary material available at 10.1007/s00192-020-04638-1.

## Introduction

Pelvic organ prolapse (POP) is the abnormal downward descent of pelvic organs, i.e., the bladder, uterus and/or the rectum, resulting in a protrusion through the vagina [1]. POP is quite common, even though many women are asymptomatic [2]. POP may be associated with a wide range of symptoms, such as the sensation of vaginal bulging, urinary and more rarely also fecal incontinence or evacuation problems, pain and dyspareunia. Patients with significant prolapse also have a significantly reduction in their quality of life [3].

Several risk factors for the later occurrence of POP have been named. The most important ones are parity, pregnancy, obesity and aging [1]. Its long-time course and the complex and multifaceted nature of this disorder make it difficult to study the condition clinically. As part of the International Urogynaecological Association’s Consultation (IUC) initiative, the Committee drafting a report on the pathophysiology of POP decided to review the literature on animal models with that perspective. Animal models are convenient as they allow for complex experimental design or discounting an abundance of interfering co-factors as in the clinical situation. Ideally, in these models the life events considered as risk factors in women should result in comparable structural and functional changes in the pelvic floor. Finding an optimal model is challenging, since humans are bipedal, have no tail and, in the context of pregnancy and delivery as a risk factor, the fetal head is relatively large compared to the pelvic dimensions, making vaginal delivery more traumatic compared to other species. Conversely, nearly all animals are quadrupeds, with a different pelvic floor musculature including a functional tail, and they have a different birth process [4].

There are occasional reports of naturally occurring vaginal prolapse in different animal species, including rabbits [5], sheep [6, 7], a number of nonhuman primates (NHPs) [8–10], cows [11, 12], pigs [13], dogs [14, 15], cats [16] and buffalos [17–19]. Most of the research in larger animal models has been done in sheep and squirrel monkeys, and more detail will be provided on findings in these species. This review will also list the work done in smaller species, but we do not cover genetic models. We first introduce clinicians to generic information on the species used in translational research on the pathophysiology of POP for further guidance.

### Reproduction cycle and comparative pelvic anatomy of species used in the study of risk factors for POP

The complex supportive system of the pelvic floor is assumed to be crucial to cope with the forces exerted when bipeds are standing upright. In quadrupeds the levator ani (LA) is responsible for tail movements [20]. In those animals the bulk of the body weight is oriented perpendicular to the spine. As a consequence, the main support for pelvic organs is bony in nature and formed by the pubic bones and ischia. Evolution to bipedalism shifted the load of the body weight parallel to the spine, and the spine, pelvis and hips are thought to be adapting accordingly. As a result, the pelvic floor became horizontal and critical for continence and the prevention of POP. Compared to quadrupeds, humans have a more complex pelvic floor and LA muscle complex [21].

## Rodents

The small size of rodents, the difference in posture, and the small size of the fetus make prolapse unlikely and render the rodent model not very appropriate for studying conditions that predispose one to developing prolapse. However, their ease of handling, short lifespan and relatively low cost, with fewer ethical constraints than higher species, are advantages [22].

Rodents have a predictable and short estrous cycle (mice: 2–5d; rats: 4d) and length of gestation (mice: 19–21 days; rats: 21–23 days) that make POP development less time intensive to study [23].

Anatomically, the gross connective tissue anatomy of the rodent pelvis is similar to that of humans [24]. The rodent pelvis has uterosacral ligaments that also attach the upper vagina to the lower spine. Paravaginal attachments insert on a dense band of connective tissue extending from the pubic symphysis to the lateral bony pelvis, which serves a role similar to that of the arcus tendineus fascia pelvis in humans. The similarity between the structure and function of the vaginal connective tissues in mice/rats and humans makes them a preferred model when evaluating connective tissue support. Although the LA (referred to as pubocaudalis and iliocaudalis muscles in the rat) is present in rodents, their primary function in rodents seems to be to support the tail, while the connective tissue attachments serve as the vaginal support [24]. One study compared the macro- and micro-anatomy of the round, uterosacral and cardinal ligaments of mice and rats, and they concluded that the rat pelvic floor structures are histologically more comparable to humans than those of mice [25].

## Lagomorphs

The anatomy of the rabbit vagina differs significantly from that of humans. The vagina is relatively long and consists of both an internal and external portion. The upper portion directly communicates with the uterus, has no adjacent connective tissue (unlike the cervix), is histologically more similar to the small intestine than to the vagina, and a large portion of the anterior wall of the external vagina includes the clitoris [22]. Rabbits do not have an oestrus cycle with spontaneous ovulation but require induction of ovulation via vaginal stimulation by coitus. Their gestation period is approximately 31–35 days.

One study compared the *microscopic* and *functional* anatomy of the pelvic floor muscles of the mouse, rat and rabbit using the architectural difference index as an indirect indicator of muscle force generating and moving capacity [26]. It was concluded that pelvic floor muscles of rats were the most similar to humans, followed by those of mice and rabbit.

## Sheep

Sheep are suggested as a large-animal alternative to NHPs [27]. They are not that expensive, available in large numbers and are often used in reproductive medicine studies [22]. Their oestrus cycle is 17 days, which is more similar to that of humans than rabbits, and their average gestation lasts 147 days. Ewes may have prolonged labors with relatively large fetuses and frequent dystocia [28]. Ewes may have antepartum cervico-vaginal prolapse (1% to 15% in specific flocks) [7, 29]. Its etiology is not well described. Several authors describe signs of milder forms of mid- and lower-vaginal descent *following* pregnancy and delivery [27, 30–37].

The dimensions of the ovine and human vagina are similar in both length and diameter [38]. Additionally, the ovine pelvic architecture relies on three levels of support, similar to those detailed by DeLancey in women [38, 39]. Sheep also have a LA complex and coccygeus muscles but have a different shape and orientation of the pelvis, and they lack sacrospinous ligaments and internal obturator muscles. On histology, the ovine vagina has four layers that are similar to those in the human vagina and a nearly comparable estrogen receptor distribution [38].

## Non-human primates (NHPs)

NHPs have histologic, hormonal and anatomical similarities to humans [40, 41]. The reproductive cycle, process of gestation/parturition, large head-to-pelvic outlet ratio [42] and hormonal effects on the pelvic organs resemble those of humans [22]. NHPs also have LA muscles consisting of the iliocaudalis (IC), pubocaudalis, and puborectalis muscles, which have analogous functions to the iliococcygeus, puboccygeus, and puborectalis muscles in humans. NHPs can develop vaginal prolapse [40, 43]. Disadvantages as an animal model include the long pregnancy and time it takes to develop spontaneous POP, the cost of maintenance, the level of expertise needed to handle them and obviously ethical constraints. We identified studies involving rhesus macaque and squirrel monkeys and baboons. Squirrel monkeys are best studied. Pierce et al. showed that female squirrel monkeys have similar intrapelvic skeletal muscular anatomy to humans and that the LA nerve originates from the S2 spinal root, yet without innervation from the pudendal nerve [41], similar to humans [44]. Their gestation is 153 days, and they have disproportionately large fetuses compared to the maternal pelvic outlet (newborn pups have a weight that is 17% of that of their mothers, compared to 8–10% in other primates [45]). Also, labor lasts long (~12 h). Moreover, when sitting their pelvis is above the ground, hence not supported and increasing the strain on it [46]. The frequent stress applied to the pelvic floor may put them at higher risk for developing prolapse than humans [22].

## Materials and methods

### Protocol and registration

This review was structured based on the guidance provided in the Preferred Reporting Items for Systematic reviews and Meta-analyses (PRISMA) statement. The research question was: “What animal models for POP are available, and what have they learnt about the relationship between aging, menopause, labour and delivery and POP?”

### Information sources, search strategy

A complete computerized literature search was conducted using MEDLINE (PubMed), Embase and the Web of Science including all studies without date and language restriction up to 15 March 2020. The electronic search strategy included both Medical Subject Headings (MeSH) and keywords (Appendix 1). Endnote X8.2 (Clarivate Analytics, Philadelphia, PA, USA), Rayyan QCRI and eventually a manual search was used to eliminate duplicate reports. Duplicates were divided into type I (duplicates among different databases) and type II (duplicate publications in different journals/issues) duplicates. Reference lists of original articles and topic-related reviews were checked manually to identify further relevant articles.

### Eligibility (studies selection, inclusion and exclusion criteria)

Two authors (MGMCMC and LH) independently screened the abstract, title, or both, of every record retrieved to determine which study should be assessed further. This was conducted using the Rayyan technology platform, Rayyan QCRI. Any discrepancies were solved through consensus. Eligible studies were those in any experimental animal in which POP was studied, either naturally occurring or provoked. Studies reporting the effects on the vagina, its support apparatus and the LA complex (or its equivalent) were included. Only articles published in English were considered. Studies reporting only qualitative outcomes, genetic models or without a proper control were not included. Review articles, case reports, commentaries, letters to the editor and unpublished articles (i.e., conference abstracts) were excluded. Since we aimed to study only the updated knowledge of animal models for POP research, articles published before than 1999 were also excluded.

### Screening methods and data extraction

All potentially relevant articles were assessed as full-text and checked for agreement. The information from these studies was tabulated according to the model, affected tissue, outcome measures and the results.

Given the heterogeneity of the included study designs and outcome measures, it was not possible to conduct a meta-analysis. Instead, all studies were appraised for reporting as a narrative review.

SYRCLE’s risk of bias tool [47] was used to assess the risk of bias in the included studies. This tool, based on the Cochrane Collaboration RoB Tool [48], aims to assess methodologic quality and has been adapted to aspects of bias that play a role in animal experiments. SYRCLE’s risk of bias tool consists of a domain-based instrument with ten items related to six types of bias: selection bias, performance bias, detection bias, attrition bias, reporting bias and other biases. These ten items are organized in subitems in the form of questions that support a “Yes,” “No” or “Unclear” answer. “Yes" refers to low bias with low risk; "No" refers to high bias with high risk; "Unclear” means the degree of risk is uncertain. Assessments were done by two independent reviewers, and disagreements were resolved through consensus-oriented discussion or by consulting a third person.

## Results

**Fig. 1:**
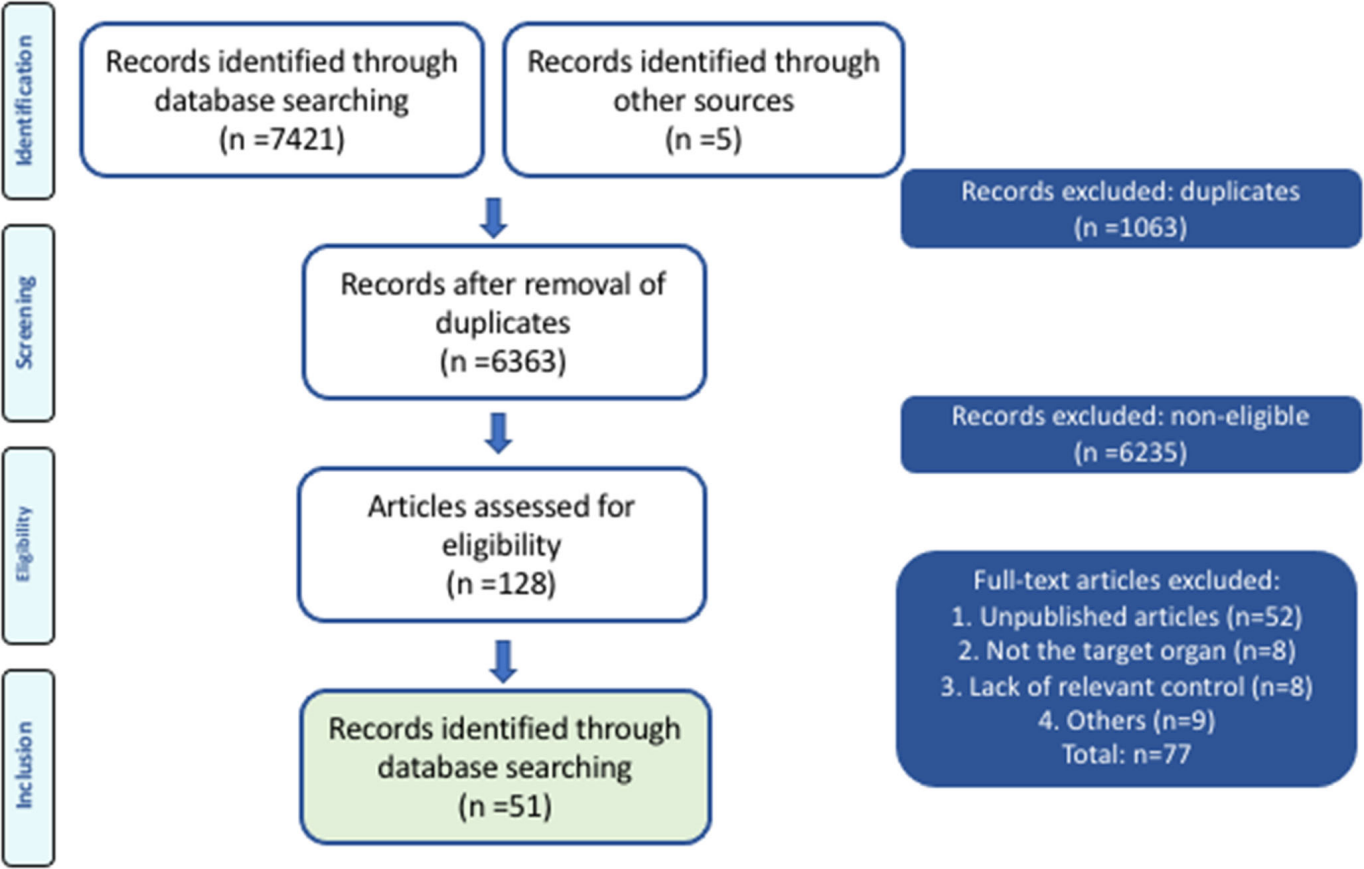
PRISMA flowchart depicting the pathway for selection of all included studies

A total of 7421 studies were identified through the search strategy, and 5 were identified through the references. After removal of duplicates, 6363 studies were screened by title and abstract. Of these, 6235 were excluded as they failed to meet the inclusion criteria. Of the 128 articles assessed for eligibility, 77 were excluded for the following reasons: unpublished articles (*n* = 52), no assessment of the vagina or pelvic floor muscles (PFM) done (*n* = 8), lack of relevant controls for the risk factor under study (*n* = 8), the studies involved only comparative anatomy (*n* = 3), the study involved male animals (*n* = 1) or not an in vivo model (*n* = 5). Eventually, 51 studies were included, and their content is summarized in Fig. 1. Bias assessment was done according to SYRCLE’s tool. Results are displayed in Table 1 for each individual study. Table 2 summarizes the findings for the different models in terms of passive biomechanics, active contractility testing, morphologic and biochemical changes.

**Table 1:**
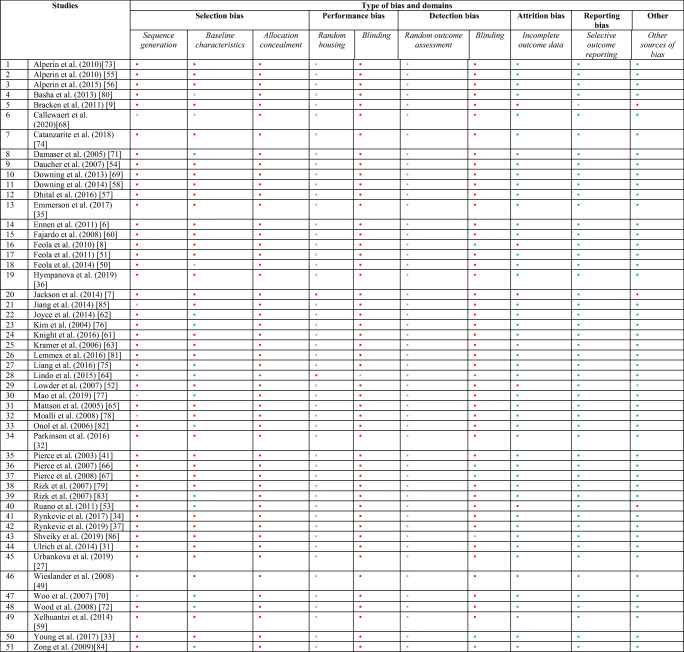
SYRCLE’s tool for assessing risk of bias. SYRCLE’s risk of bias tool consists of a domain-based instrument with ten items related to six types of bias: selection bias, performance bias, detection bias, attrition bias, reporting bias and other biases. These ten items are organized in subitems in the form of questions that support a “Yes,” “No” or “Unclear" answer. “Yes" refers to low bias with low risk; "No" refers to high bias with high risk; "Unclear” means the degree of risk is uncertain. For easier orientation, answers at each domain are represented by color dots as follows:  Yes,  No,  Unclear

**Table 2:** Summary of findings of active and passive biomechanical testing and structural analysis of the vagina in the different species used as a model for POP. Results are displayed as a comparison to nulliparous females

	Species	Passive biomechanics	Active biomechanics	Collagen content	GAG content	Elastin content	Muscularis layer
Pregnancy	***Rat***	Increase compliance [50–52] and decrease tensile strength [51, 52]	Decreased contractility [51]	Decreased fiber aerea [54]	Decrease GAGs [53]	Upregulation of genes involved in the elastin metabolism [69]	__
	***Sheep***	Increase compliance and decrease tensile strength [30, 34, 37]	__	Decrease [30, 34, 37]	__	Increase [30, 34, 37]	Increase [30, 34, 37]
Spontaneous vaginal birth (primiparous)	***Rat***	Initially increase compliance, which returned to normal 2w after delivery [58]	__	__	__	__	__
	***Sheep***	Increased compliance [27, 36, 61] and decrease tensile strength [61]	*Ambiguous* No difference [36] Decrease to KCl (distal vagina) [27]	Decreased [27, 36]	__	Increased [27, 36]	Decrease [27]
Spontaneous vaginal birth (multiparous)	***Rat***	Increase compliance [58]	__	Decrease [57]	__	Tortuosity of the elastin fibers decreased [69]	Dissociation of collagen fibers with smooth muscle [57]
	***Rabbit***	__	__	Decrease [59]	__		Decrease [59]
	***Sheep***	*Ambiguous* No effect [37]or Increase compliance [32, 34, 35]	__	*Ambiguous* No difference [36]; decrease [35,37,34]or increase [30]	__	*Ambiguous* Decrease [30]or increase [34–36]	*Ambiguous* *No effect* [30]; Increase [37]or decrease [35]
	***NHP***	Increase compliance and decrease tensile strength [8]	__	Loss of collagen alignment [8]	__	__	__
SVD	***Rat***	Initially increase compliance, which returned to normal [69] increase compliance and decreased tensile strenght [73]	Initial hype- and later hyposensibility to carbachol (muscarinic receptors) [68]	No differences in connective tissue [68] and decrease collagen I/V ratio [73] on the long term	Increase [69],	Initial increased tortuosity of the elastin fibers, which normalized afterwards [69]	Decrease (disruption of smooth muscle layer) [68, 73]
Iatrogenic menopause	***Rat***	Initially increase compliance and decrease tensile strenght [75, 78] and later decrease compliance [77]	*Ambiguous* for the vaginal region No effect in the proximal vagina. Distal vagina: decrease to KCl and phenylephrine. [82] Proximal vaginal: decrease to KCl [80]	Initially no difference [75], later increase in the mature and decrease in the immature collagen [77]	No difference [75]	__	No difference [80] or decrease [77, 82]
	***Rabbit***	Increased compliance in adults OVX but not adolescents OVX [81]	No difference [76]	Increase in adolescents OVX [81]	No difference [81]	__	Decrease [76]
	***Sheep***	No effect [36]	Decrease contractility in the middle vagina of OVX multiparous compared to intact multiparous [36]	Increase compared to intact multiparous [36]	__	Decrease only compared to intact multiparous [36]	No effect [36]
Aging	***Mouse***	__	__	__	__	Downregulation of genes involved in the elastin metabolism [85]	__
	***Rat***	No effect [78, 86]	__	__	__	__	__

### Pregnancy and parity

Effects of pregnancy and parity on the vagina and the pelvic floor muscles were reported in 30 studies: 1 in mice [49], 9 in rats [50–58], 2 in rabbits [59, 60], 11 in sheep [6, 7, 27, 30, 32–37, 61] and 7 in NHPs [8, 9, 62–66] (Supplementary Table 1). The effect of *pregnancy* was reported in 13 studies (mice = 1 [49]; rats = 7 [50–56] and sheep = 5 [6, 7, 30, 34, 37]). Across species, pregnancy has a measurable effect, though that was most extensively documented in rats and sheep. Vaginal compliance increases during pregnancy [30, 34, 37, 50–52]. This is paralleled by microscopic and biochemical changes, i.e., decreased collagen [30, 34, 37, 53, 54] and increased elastin [30, 34, 37]. The changes observed are considered as adaptations to prevent later damage caused by the passage of the fetus. The *first delivery* has an obvious effect. Most studies report an increase in compliance [27, 36, 61] and loss of tensile strength [61] of the vagina. Structurally, after the first delivery, collagen is reduced and elastin is increased [27, 36]. The additional effects of subsequent deliveries are variable and not similar in all studies. In some studies in multiparous sheep, the initial effects of delivery actually recover [35, 36]. Available studies do not quantify the effect of age at the time of delivery in either rats or in sheep.

### Denervation

Squirrel monkeys display POP under the form of cystocele following delivery, which is incremental with the number of deliveries. POP is facilitated by neurectomy, though neurectomy itself does not cause POP [41, 67] (Supplementary Table 2).

### Simulated vaginal delivery

Rats are the only species in which effects of delivery were studied by simulating this event (Supplementary Table 3). Eight studies reported the effects on the vagina [53, 58, 68–73] and one on the pelvic floor muscles [74]. SVD induces structural, active and passive biomechanical changes, which in rats partly heals relatively quickly, but without complete recovery of the muscular layer [55, 68, 74], microvasculature [68] and biomechanics [55, 68]. However, SVD does not lead to POP.

### Iatrogenic menopause

Changes induced by ovariectomy (OVX) in rats, rabbits and sheep were described in 11 studies [36, 75–84] (Supplementary Table 4). The effects depend on the age when OVX is done and the interval to measurement. Menopause initially increases compliance [75, 78, 81]; however, later on the vagina becomes stiffer in rats [77]. There is no effect in sheep [36]. OVX induces atrophy of the vaginal epithelium [36, 76, 82], an increase in collagen [36, 77, 81] and decrease in elastin [36] and muscularis [76, 77, 82]. These effects are reversible by administration of hormones [75, 76, 78, 80, 83, 84]. OVX itself does not lead to prolapse.

### Aging

Five studies investigated the effect of aging on the vagina: one in mice [85], three in rats [78, 79, 86] and one in the baboon [65] (Supplementary Table 5). All of them reported the effect of *natural* aging, except one study in mice, which also reported the effect of busulfan, a drug that accelerates aging. In mice and rats, there is no measurable biomechanical effect of age; however, there are arguments for a change in elastin metabolism [85].

## Discussion and conclusion

### The effect of pregnancy and parity among species

#### Large animal models

*Squirrel monkeys* are non-human primates that are considered good models for studying the pathophysiology of pelvic floor dysfunction, including POP. Several risk factors for POP have been studied in this species, such as the relation with the pelvic outlet diameter, age, parity and body weight [62]. Of these, only *parity* was strongly correlated with the development of bladder descent (defined as 7 mm below the bony pelvis). The effects of pregnancy and delivery were studied in detail, using magnetic resonance imaging to assess the anatomy of the pelvic floor muscles and width of the bony pelvic outlet and measure bladder neck descent [9, 64]. The muscles studied were the levator, obturator internus and coccygeus, which are all considered relevant to pelvic floor support. In particular, the coccygeus muscle was directly affected by the passage of the fetal head during delivery [9, 64]. Immediately postpartum, there is a *reduction* in levator and obturator internus volume, but this effect was *similar* following vaginal and abdominal delivery. The reduction would be the consequence of relative atrophy due to decreased physical activity of these muscle groups during pregnancy, hence not related to the delivery itself. Vaginal delivery was associated with a (temporary) increase in volume in the coccygeus muscle that was not observed after cesarean section. This would be indicative of tissue edema and hence be an indirect sign of trauma by passage of the head. At 3 to 4 months after delivery, no permanent anatomical changes are visible in the pelvic floor muscles anymore [9, 64]. On the other hand, the bladder neck position is lower immediately after vaginal delivery and even more 3–4 months postpartum [9, 64] . This was associated with an increase in the width of the pelvic outlet [64]. Remarkably, the extent of the descent and the width of the pelvic outlet were similar 3–4 months postpartum, whether delivery was vaginal or by cesarean section [64]. The authors thought that this was due to permanent structural changes in the supportive pelvic floor ligaments and connective tissue induced during pregnancy (hence not birth). Therefore, in squirrel monkeys, cesarean section does *not* prevent changes induced by pregnancy and delivery. In another study, the presence of POP did not coincide with any gross anatomical differences in the pelvic floor muscles [63], but microscopically the myocytes of squirrel monkeys with POP were larger. No increase in apoptosis, disruption or atrophy were present [66]. We did not find information regarding the compliance and structural changes of the vagina in squirrel monkeys with POP.

Some, but not all, primates develop POP after (multiple) deliveries. For instance, multiparous Rhesus macaques spontaneously develop descent of the cervix and posterior fornix—yet no other compartment [8], whereas multiparous baboons do not [65]. In the macaque, vaginal compliance increases and tensile strength becomes less, in analogy to what is described in sheep and rats [34, 35, 69]. Microscopically, the occurrence of POP coincided with a loss of collagen alignment but no difference in collagen subtypes.

*Sheep* are also said to develop “spontaneous” POP in the context of pregnancy and delivery [32, 33, 38]. They can develop impressive degrees of prolapse *before* birth. This suggests that, in some animals, structural effects occur during pregnancy, eventually leading to POP, though this may be a degree of laxity that is probably not what clinicians would consider as representative for what is a typical presentation in women. Excessive weight gain during pregnancy, living on a steep terrain as well as having twins (RR:5.0) and triplets (RR:11.0) are risk factors for *antepartum* POP [7]. In sheep with antepartum prolapse, there were no differences in progesterone or estradiol levels compared to those who did not [6]. At the gene expression level, sheep with antepartum POP display downregulation of collagen I [6], which is important in structural support. They also display hyperplasia of the vaginal epithelium, though they did not have elevated circulating estrogens [6]. The ewes with POP also had lower estrogen receptor alpha levels. This is different in premenopausal women with POP, who have been reported to have lower estrogen levels associated with less receptor expression [6].

Properties of pelvic floor structures can also be characterized by passive mechanical testing, which describes the relationship between stress and strain, and measurement of the disruption force. In sheep, pregnancy induces an increase in compliance and loss of tensile strength of the vagina [30, 34]. Other studies have documented the effects of a single or multiple delivery compared to the status in virgins. Primiparous ewes have an increased vaginal compliance and lower tensile strength. Also, this seems to be the case for multiparous females, except in two studies [36, 37]. One of these studies [33] is interesting, because it introduces a quantitative POP system by measuring *spontaneous* displacement of a point 3 cm above the introitus, both anterior and posterior in the vagina, as well as a point above the urethra. Primiparous sheep displayed “displacement” in the lower areas and multiparous also of the point higher up in the vagina. The authors concluded that sheep display “similar regions of weakness” as in humans [33]. In another study, the gross anatomic changes were documented in primi- and multiparous sheep. Primiparous sheep displayed an increased width and length of the vagina, which again returned to normal in multiparous sheep [36]. In another study in multiparous sheep, thinning of the vaginal wall was documented [35]. In conclusion, in sheep, pregnancy and delivery induce compliance and anatomical changes, but there is an inconsistency on whether multiple deliveries are causing incremental changes, and it remains unstudied whether changes observed after delivery did occur during pregnancy and/or recover in between pregnancies. In future experiments, longitudinal observations should be included. This could be easily done with an elegant, purposely designed device permitting non-destructive in vivo compliance measurements [35].

The findings at the microscopic level in sheep are less clear. *Pregnancy* induces an increase in elastin fibers, thickening of the muscularis layer and markedly less dense collagen in all vaginal layers compared to virgin non-pregnant ewes, without change in vascularization in the lamina propria [30, 34, 37, 52]. The biochemical findings confirmed the increase in elastin but not the collagen changes [30]. The discrepancy between morphology and biochemical findings is not always easy to interpret. The long-term findings *after one or more deliveries* are quite conflicting. For instance, there are studies that did not document a change in collagen [35, 61], and others reported a decrease [34, 36, 37]. The same goes for elastin (increased [34–37] and decreased [30]) or the thickness of the muscularis (increased [34, 37] vs. decreased [35]). One study also biochemically measured the total collagen, its subtypes, elastin and glycosamin glycans (GAGs). The results do not all parallel the morphologic findings, making the interpretation difficult [30].

In conclusion, the overall impression is that, in sheep, *pregnancy* modifies the tissues such that the vagina becomes more compliant. This suggests that structural and functional adaptations may account for the ability of the vagina to withstand “supraphysiologic” strains during parturition without injury [30, 34, 37]. Another method to study the mechanical properties of the vagina is by *active* contractility testing. Some consider this as a “functional” test. It measures the ability of the vaginal smooth muscle layer to contract when exposed to agents like KCl or K^+^ or by electric field stimulation. The response is proportional to the amount of smooth muscle tissue present. One can also stimulate the muscle via its innervation by adrenergic agents such as phenylephrine, epinephrine or norepinephrine, or by cholinergics like carbachol. Active biomechanical properties are only rarely reported in sheep. One study demonstrated no measurable long-term impact of parity on the active contractility of the vagina in primiparous or multiparous [36].

#### Smaller species

Lower species allow the study of pregnancy and delivery in much more detail, though as they are smaller the relevance of it may be more questionable. Again, all studies agree that *pregnancy* induces an increase in compliance and loss of tensile strength [30, 34, 37, 50–52, 55]. These changes recover after vaginal delivery, but the exact time point and level to which this occurs is inconsistent. In some this happens within 1 week [50], in others within 4 [51, 52], yet still incompletely [55]. In one study also multiparous animals were studied. They also display an increase in compliance compared to virgins. However, virgin rats were 4 months old and multiparous 9 months old [69]. Therefore, in that study it was not possible to conclude if the changes were due to parity or age, though other experiments have shown that age does not have an effect on vaginal biomechanics [78, 86]. The functional (active) response of vaginal smooth muscles during pregnancy and after delivery was tested as well. An increased sensibility to KCl was observed during late pregnancy, which did not return to pre-pregnancy values 4 weeks postpartum [51]. Another study investigated the function of the perineal and pelvic muscle following electric stimulation in rabbits. Multiparous females had lower switch and tetanic tension force than virgin animals [60].

In rats, morphologic changes were also characterized. During late pregnancy the vagina lengthens, to normalize by 4 weeks postpartum [51]. On the other hand, the distal vagina is the widest at 1 week postpartum [50]. Moalli’s group documented pregnancy-induced microscopic changes, demonstrating an increase in the thickness of the muscularis layer, a decrease and loss of organization and orientation of the collagen fibers, and an increase in elastin [54]. Morphologically, the smooth muscle phenotype changes from a quiescent to a proliferative and synthetic one [54]. Alperin’s group described an increased muscle fiber length in the M. coccygeus, iliocaudalis and pubocaudalis in rats during late pregnancy [56]. This effect was associated to the adaptations of the PFM to have a protective effect against damage from large mechanical deformations likely occurring during parturition.

In one study, also GAG levels were assessed. GAGs play an important role in the properties of the extracellular matrix. In that study pregnancy induces a remarkable drop in GAG levels. Changes *after* delivery were documented in multiparous rats in two studies [57, 69]. There was a signature of collagen fiber dissociation with the smooth muscle and change in the density of collagen fibers [57]. In the study focusing on changes in vaginal GAG levels, a comparison in GAG levels after vaginal delivery and cesarean section was made. Vaginal delivery induces a deeper short-term drop in GAG levels than after abdominal delivery. On the long term (3 months), however, the GAG levels increase to far above pre-pregnancy levels. The study is unclear about whether the difference between abdominally and vaginally delivered rats is significant [53]. The design of that study is an interesting one to dissect out the effects of pregnancy, delivery and the severity of birth trauma (the authors also simulated deliveries). Changes in the extracellular matrix metabolism were also studied in other species. For instance, in mice, a downregulation of Mmp2 and Mmp9 during pregnancy and immediately postpartum has been described [49].

### Models that study the effects of vaginal birth

#### Denervation

Denervation,, e.g., as in congenital birth defects such as spina bifida, or by traumatic delivery, has long been tied to the occurrence of POP. Denervation has been simulated in animal models, including for the study of POP. In rats, the effect of pudendal nerve crush has been widely studied, yet typically in the context of simulating urinary and fecal incontinence. Also experiments in squirrel monkeys involved denervation in the pudendal and LA nerve area. Atrophy of the M. pubocaudalis and iliocaudalis could only be induced by neurectomy of the LA nerve [41]. In other words, unlike in humans, the pudendal nerve does not innervate those muscles. The interesting part of that study was that the long-term effects of induced muscle atrophy were also documented [67]. Nulliparous squirrel monkeys that had undergone bilateral LA neurectomy did not develop bladder descent within 2 to 3 years. Apparently, despite muscle atrophy, other support structures prevent POP to develop. In the animals that had undergone neurectomy, who became pregnant and delivered vaginally, all developed bladder descent. Two animals that died from obstetrical complications underwent necropsy, which revealed fibrosis, muscle atrophy and fatty replacement. In conclusion, denervation does not cause POP by itself, but may contribute to the onset of vaginal prolapse in animals that delivered vaginally. The effect of pregnancy or of cesarean section alone was not studied.

#### Simulated vaginal delivery

Researchers have also documented the effects of *simulated* vaginal delivery in rats on both the vagina and pelvic floor anatomy as well as functional changes in vaginal function and urinary continence. Those experiments were conceived to provoke more changes than what is spontaneously occurring, since rats have a much smaller fetal head-to-pelvic outlet ratio than humans. One way to achieve this is by vaginal distention (VD). For that purpose, a balloon is inserted in the vagina and inflated with different volumes (2.5–5 ml) and for a given duration (1–6 h). This causes both mechanical stretch as well as hypoxia. Unfortunately, there is no standardization of either the model or readouts. The importance of standardization becomes obvious in an experiment made by the group of Alperin et al. They documented the dose-response curve of increasing degrees of VD on the pelvic floor muscles (M. coccygeus, ileocaudalis and pubocaudalis). They compared the effects to that of *spontaneous vaginal* delivery. The outcome measure of this experiment was the change in microstructure of the pelvic floor muscles (fiber and sarcomere length) to identify hyperelongation of the sarcomere as a primary cause of mechanical injury and resultant muscle dysfunction. A filling volume of 3 ml distention mimics the effects of spontaneous vaginal delivery [74]. They demonstrated that delivery acutely stretches the myofibers, distorts the Z-lines and misalignment of adjacent sarcomeres, and increases sarcomere length. The changes were proportional to the distention volume used for simulation. The changes were also different when delivery was simulated in animals that were pregnant versus rats that were not, i.e., the injury was worse in the latter scenario. In other words, pregnancy has an attenuating effect on structural muscle changes, in particular in the M. coccygeus and M. pubocaudalis.

In two studies the changes in passive biomechanics of the vagina were investigated. VD increases vaginal compliance 2 days after injury; however, normal properties were observed 2 weeks after delivery [69]. In another study with a higher filling volume, the increased compliance persisted up to 4 weeks after VD [73]. VD also induces anatomical changes. Macroscopically, the vagina was 20 to 50% wider following VD [73]. Microscopically, VD causes a combination of hypoxia-induced and stretch injury in the vagina. Significant hypoxia in the epithelial layer and a lower level of hypoxia in the muscularis were observed 1 h after VD [71]. VD induces a disruption of the fibromuscular layer of the vagina which persists until 4 weeks after delivery [68, 73]. On immunochemistry, VD induced an increase in the collagen I/V ratio, but not in the I/III ratio, 4 weeks after delivery and tortuosity of elastic fibers 2 days after VD [69]. By 2 weeks, the elastic fibers appeared normal [69]. As in spontaneous vaginal delivery, VD increase GAG levels in the vagina within 3 months, following an initial decrease 4 days after VD [53]. In conclusion, VD induces extracellular matrix (ECM) production after the remodeling phase. Though immediately after VD there are no measurable differences in mRNA expression of genes related to inflammation or hypoxia [72], later on (by 24 h) there is upregulation of MCP-3 and SDF-1, which are known markers of mobilization and homing of stem cells [70].

Another strategy is to induce nerve damage, which in rats typically is achieved by pudendal nerve crush (PNC). We could not find studies that document the effect of PNC alone on the vagina. The combination of VD and PNC creates longer lasting functional and anatomical effects. The downstream pelvic floor dysfunction effects can be measured at different levels, such as urethral and anal sphincter function, but also in the vagina. In several experiments, both strategies (VD and PNC) were combined, though only one focused on vaginal changes [68]. Active contractility was tested in the vagina at 1, 2, 3 and 6 weeks after VD + PNC. Functionally, there seems to be a given time course, with an initial increased response to carbachol at 2 and 3 weeks and decrease at 6 weeks. The combination of VD and PNC induces a loss of microvasculature at 1 week, without recovery by 6 weeks. As following VD only, there was disruption of the muscle layer, which was eventually replaced by scar tissue. In the vagina, there was upregulation of smoothelin (smooth muscle regeneration), rock 1 (fibrosis) and muscarinic receptor 2 (acethylcholine receptor) at 3 days and downregulation of caldesmon (smooth muscle regeneration) and upregulation of collagen III at 7 days. The authors concluded that initially the vagina has a hypersensitivity denervation, which is characterized by an increase in the number of receptor sites, in an effort to maintain synaptic homeostasis, following neurotransmitter depletion. This was in agreement with the initial upregulation of muscarinic receptors 2 and its normalization later on, which coincided with a reduced sensitivity to carbachol. Another hypothesis was the impairment of the contractility by the process of fibrosis, since an increased collagen I/III ratio also took place 6 weeks after injury [68]. The initial upregulation of smoothelin may be seen as an attempt to regenerate the vaginal smooth muscle; however, it seems that this process was impaired since a downregulation of caldesmon was also observed at 7 days.

### Iatrogenic menopause

OVX is the standard surgical procedure to investigate the effect of menopause in experimental animals, because the species used for the study of POP do not develop spontaneous menopause. In rats, OVX reduces the stiffness of the vagina in young (4-month-old) rats after 8 weeks [75, 78]; however, no such effect was observed when OVX was performed in old (9-month-old) rats [78]. Another study reported a significantly increased stiffness 16 weeks after OVX in young rats [77]. In conclusion, in the longer term OVX in rats induces an increased stiffness of vaginal tissues. In rabbits, there was also an age-dependent effect. In adult rabbits, OVX led to an increased compliance of the medial collateral ligaments (no measurements in the vagina were done), but not in adolescent rabbits [81]. In multiparous sheep, no change was seen 160 days after OVX [36].

The currently available findings on active contractility are not consistent. In the distal [82] and proximal vagina of rats [80] and middle vagina of sheep [36] OVX decreased active contractility. This is not the case in rabbits [76].

Another outcome measure is vaginal morphology. Macroscopically OVX induces thinning of the vaginal wall in rats and rabbits [76, 80], and in sheep the vagina gets shorter and narrower [36]. Atrophy of the epithelial layer is a consistent finding across species, including rats, rabbits and sheep [36, 76, 77, 80, 82]. This coincides with thinning of the layer of glycogen-containing cells [36]. There is also atrophy of the muscularis [36, 76, 82]. One study in rats did not, but this may be due to the short interval between OVX and readout (3 weeks) [80]. An increase in collagen has been documented morphologically in both rats and sheep [36, 77], with proportionally more mature collagen at 16 weeks in rats [77]. This effect was not present in rats 8 weeks after OVX [75]. In sheep, a decrease in elastin was also reported [36]. At the protein level, in rats OVX induces upregulation of mature collagen and downregulation of immature collagen [77] and upregulation of a key collagenase (Mmp13) [84] and of urogenital aging markers (isomyosin and P27k^ip1^) [79]. Conversely, downregulation of gene expression of muscle markers SM1 and caldesmon has been reported as well [80]. Hormonal replacement reverses most of the changes caused by OVX in rats, rabbits and sheep [75, 76, 78, 80, 83, 84].

Overall, the biomechanical response in experimental animals is dependent on the age when OVX is induced and the interval to measurement. In the longer term, the vagina becomes stiffer, and this is associated an increase in collagen.

### Aging

Both accelerated aging and natural aging led to a drop of 62% and 44% of estradiol compared to young mice, demonstrating an intertwinement between hormonal changes and advancing age [85]. Lower levels of estradiol were also observed in aging rats [86]. Both accelerated and natural aging induce downregulation of gene and protein expression of Lox3 and Lox4, which play a role on the synthesis of elastic fibers [85]. Again, in rats, aging did not influence the compliance of the vagina [78, 86]. However, aging seems to have an effect on the healing process of the vagina. Thirty days after injury, old rats regain only 15% of its original strength and compliance whereas young rats recovered for 60%. This was associated with delayed and long-lasting expression of MIF (macrophage response). In baboons, aging did not coincide with more signs of POP [65].

### Methodologic comment and recommendations

Most of the reviewed articles had more than one methodologic shortcoming. Most studies did not provide proper animal randomization and information on housing and whether this was randomized. Animals differed in the baseline characteristic such as weight, sex or age. Furthermore, blinding of the researchers taking care of animals as well as researchers analyzing the outcomes was mostly missing. Almost all studies did not use power calculation. Another problem is the heterogeneity of the methodology used, for example, in the view of biomechanical testing, where a broad spectrum of methodology is used. Future studies should avoid previously mentioned shortcomings by conducting well-designed, powered and blinded studies with homogeneous animal subject and methodology.

## Conclusion

Several animal models have been used in the study of the pathophysiology of POP, each with its own purpose, merits and limitations. In several species there are measurable effects of pregnancy, delivery and iatrogenic menopause, but there is not a single uniform pattern. Only squirrel monkeys develop clinical POP spontaneously.

## Supplementary Information

ESM 1 (DOCX 110 kb)
