# Supplementary material for: Animal models for pelvic organ prolapse: systematic review
Source: Int Urogynecol J. 2021 Jan 23;32(6):1331–44. doi: 10.1007/s00192-020-04638-1 (PMC8203535; doi:10.1007/s00192-020-04638-1)
Supplement: Supplementary file 1 — (DOCX 110 kb) [file 192_2020_4638_MOESM1_ESM.docx]

**Supplementary data**

**Animal Models for Pelvic Organ Prolapse: Systematic Review**

*Marina Gabriela M. C. Mori da Cunha^1^*^,2^, *Katerina Mackova^1,2,3^, Lucie Hajkova Hympanova^3,^, Maria Augusta T. Bortolini^4^, Jan Deprest^1,2,5*^*

^1^ Centre for Surgical Technologies, Group Biomedical Sciences, KU Leuven, Leuven, Belgium

^2^ Department of Development and Regeneration, Woman and Child, Group Biomedical Sciences, KU Leuven, Leuven, Belgium

^3^ Institute for the Care of Mother and Child, Third Faculty of Medicine, Charles University, Prague, Czech Republic

^4^ Department of Gynecology, Sector of Urogynecology, Universidade Federal de São Paulo, São Paulo, SP, Brazil

^5^ Pelvic Floor Unit, University Hospitals KU Leuven, Leuven, Belgium

*corresponding author Department of Development and Regeneration. Center of Surgical Technologies UZ Herestraat, 49 B3000 Leuven, Belgium. email: [Jan.Deprest@uzleuven.be](mailto:Jan.Deprest@uzleuven.be) Tel: 32 1634 4215 - Fax: 32 1634 4205

Supplementary table 1: Studies reporting the effect of pregnancy and parity in relevant species.

| **Reference** | **Sp. and relevant groups** | **N per group and time point** | **Tissue investigated** | **Outcome measures** | **Main effects** |
| --- | --- | --- | --- | --- | --- |
| Wieslander, 2007 [49] | Mouse (age unknown); 1. Virgin, 2. Early pregnancy, 3. Late Pregnancy, 4. 2-48h PP, 5. 1w PP, 6. 2w PP | N=3 | Vagina | Gene expression: Mmp2, Mmp9 and Mmp12 and their inhibitors | During pregnancy and immediately PP: downregulation of MMP2 and MMP9 and upregulation of elastase inhibitor;  48h PP upregulation of MMP2, MMP9,decrease of protease inhibitors. |
| Daucher, 2007 [54] | Rat (age unknown); 1. Virgin, 2. Mid pregnancy (12-14d), 3. Late pregnancy (20-22d), 4. Immediately PP 5. 3w PP | N=4 | Vagina | Histology: Gomori Trichrome, Toluidine Blue | Midpregnant and late pregnant: an increase in the number of papillae, the epithelium became mucified, an increase in matrix deposition in the subepithelium and muscularis |
|  |  |  |  | TEM - morphology of smooth muscle cells and collagen fibril | Pregnancy decrease collagen fiber area and SMCs transforms from a quiescent and contractile SMCs to proliferative and synthetic phenotype. SMCs became diffusely spaced and surrounded by ECM.  Immediately PP collagen fibers lost organization and orientation and the SMCs reduces the synthetic profile and normalized 3w PP. |
| Lowder et al., 2007 [52] | Rat (3m): 1. Virgin, 2. Mid pregnancy (d13), 3. Late pregnancy (19-21d), 4. Vaginal delivery (Immediately 0-4h and 4w), 5.CS (Immediately and 4w) - primigravid | N=8-13 | Vagina and supportive tissue complex | Passive biomechanics | Pregnancy decrease stiffness and ultimate load at failure; maximal distension at the time of delivery; normalized at 4w PP |
| Alperin et al., 2010 [55] | Rat (age unknown); 1. Virgin, 2. Late pregnancy (17-20d), 3. 4w PP | N=7 -10 | Vagina | In vivo passive biomechanics | Compliance of the vagina increases during pregnancy and it recovered partially 4w PP but not until virgin compliance |
|  |  |  |  | Vaginal dimensions (GH, TVL and vaginal diameter) | Genital hiatus, total vaginal length and vaginal diameters did not differ between the groups |
| Feola et al., 2011 [51] | Rat (3m); 1. Virgin, 2. Mid Pregnancy (14-16d), 3. Late Pregnancy (20-22d), 4. Immediately PP (0-2h), 5. 4w PP | N=6-8 for passive biomechanics and N=5-9 for active contractility | Vagina | Passive biomechanics | Decreased compliance during pregnancy and immediately postpartum, by 4 weeks postpartum recovered to virgin levels |
|  |  |  |  | Active contractility | Contractile force decreased in mid- and late-pregnancy and immediately post- partum compared to virgin animals, 4 weeks postpartum returned to virgin levels |
|  |  |  |  | Vaginal dimensions (TVL and GH) | TVL and GH values increased from virgin to late-pregnant animals and immediately PP; values normalized 4w PP |
| Ruano, 2011 [53] | Rat (4m); 1. Virgin, 2. Virgin SVD, 3. CS, 4. CS + SVD, 5. Spontaneous vaginal delivery, 6. Late pregnancy (d20) | N=10 | Vagina, 4d or 3m after delivery or SVD | GAG biochemical analysis | Pregnancy decrease the levels of GAGs; normal delivery or SVD increase GAGs at 3m |
| Downing, 2013 [69] | Rat (age unknown); 1. Virgin, 2. Virgin SVD 3. Spontaneous delivery, 4. CS; SVD: VD 2.5ml, hanging catheter for 3h; still inflated catheter when removed | N=5-6 | Vagina, 2d and 2w after delivery or SVD | Gene expression (genes involved in elastic fiber homeostasis): Loxl-1, Fbln5, tropoelastin | Vaginal delivery, SVD and pregnancy cause upregulation of genes involved in elastic fiber homeostasis. |
| Downing, 2013 [58] | Rats (age unknown); 1. Virgin, 2. Virgin SVD, 3. Vaginal delivery, 4. SC, 5. Multiparous | N=6 per group and time point | Vagina, 2d and 2w after delivery or SVD | Passive Biomechanics | Regardless the mode of delivery, vagina have a significantly higher compliance 2d after delivery compared to virgin rats; it normalizes at 2w. In multiparous compliance was higher than in nulliparous. |
|  |  |  |  | Histology - Hart s - Morphometric analysis of elastin fibers | Regardless the mode of delivery, vagina have a significantly higher tortuosity of elastic fibers 2d after delivery compared to virgin rats; and it normalizes at 2w. In multiparous tortuosity of elastic fibers was higher than in nulliparous. |
| Feola et al., 2014 [50] | Rat (age unknown); 1. Virgin, 2. Longitudinal study: Pregnancy (15d and 18d) and PP (7d, 30d) | N=6-8 | Vagina | In vivo biomechanics of the vagina by 3D US | Pregnancy increase compliance (3.5x at 15d and 5.4x at 18d; it normalizes at 1w PP |
|  |  |  |  | Shape and size of vagina | Distal vagina has the highest difference in the cross-sectional area; which 1w PP had the largest area (58% larger than 4w PP) |
| Alperin, 2015 [56] | Rats (3m); 1. Young Virgin (3m), 2. Mid pregnancy 3. Late pregnancy, 4. Mature Virgin (6m) ,5. 4w PP, 6. 12w PP | N=10 | PFM | Histology: Morphometric analysis (fiber and sarcomere length and physiologic cross-sectional area) | Late pregnancy induces increase of fiber length in all PFM (coccygeus, iliocaudalis and pubocaudalis), no effect in tibialis. Recovery of fiber length at 12w PP. No effect in sarcomere length and cross-sectional area; |
|  |  |  |  | Total collagen | Increased during pregnancy, no decrease in coccygeus PP. |
| Dhital, 2016 [57] | Rats; 1. Virgin (11–14 w), 2. Multiparous (9–15 m), 3. 2d PP (primiparous), 4. 14d PP (primiparous) | N=4 | Vagina | Analysis of collagen by nuclear magnetic resonance (NMR) | Virgin rat tissues contained higher collagen content compared to the other three groups |
|  |  |  |  | Biochemical analysis of collagen | Multiparous rats showed higher collagen content values compared to 2 and 14 d PP |
|  |  |  |  | Histology: Van Gieson | Collagen fiber dissociation with smooth muscles in multiparous rats |
| Fajardo, 2008 [60] | Rabbit (10-14m); 1. Virgin, 2. Multiparous | N=5 | Perineal and Pelvic Muscles (Bulbospongiosus, Pubococcygeus) | Histology: Sudan Black | Multiparous have smaller cross-sectional muscle fiber area |
|  |  |  |  | Contractile properties - electrical stimulation | Multiparous have lower switch and tetanic tension force in response to electric stimulation |
|  |  |  |  | Weight of muscle | Multiparous have lighter muscle |
| Xelhuantzi, 2014 [59] | Rabbit; 1. Virgin, 2. Multiparous (4 deliveries);  Subgroups: young (9-11m) and adult (17-18m) | N=6 | Vagina | Histology: MT | Multiparous: Disorganization of the tissue components in vagina; less collagen, muscle and vessels |
| Ennen et al., 2011 [6] | Sheep (3.2 ± 1.4 y); 1. Healthy pregnant, 2. Intrapartum, 3. PP with POP | N=4-6 | Blood | Radioimmunoassay: Serum levels of Progesterone and 17β-Estradiol | No difference |
|  |  |  | Vagina | Histology: HE | POP: hyperplasia of the epithelial cells. No evidence of increased inflammation. |
|  |  |  |  | Gene expression RT-PCR: GAPDH, Mmp1, Timp1, ER-α, Col1a2 | POP: Downregulation of col1a2 and ER-α |
| Jackson et al., 2014 [7] | Sheep (mixed age): 1. Whole farm population, 2. Randomly selected ewes (2y and older) | N=36,695 | - | Epidemiologic test; risk factors of vaginal prolapse | 5x higher chance of POP with twins; 11x triplets; higher risk with weigh gain during pregnancy, access to salt and steep terrain |
| Ulrich, 2014 [30] | Sheep; 1. Virgin (1y), 2. Late pregnancy (with 3 previous deliveries; 4-5y), 3. Multiparous (3 deliveries; 4-5y) | N=3-6 | Vagina | POPq | Virgin and parous no displacement. In pregnancy, the mean displacement was 3.3 cm with traction of both vaginal walls. |
|  |  |  |  | Passive Biomechanics | Pregnant: weakest and most compliant vagina; parous: highest max stress; virgin: strongest and still compliant tissue |
|  |  |  |  | Biochemical analysis (hydroxyproline (Hyp) assay :total collagen, SDS-polyacrylamide gel electrophoresis: collagen III/I, colorimetric dimethylmethylene blue (DMMB) assay : GAG and indirect insoluble elastic tissue associated proteins (ETAP) analysis: elastin) | Parous increase in collagen and decrease in elastin compared to virgin;. Pregnant increase elastin compared to virgin. Similar levels of total collagen. No difference in collagen III/I. The virgin lower GAG in the anterior wall compared to the parous. |
|  |  |  |  | Histology: HE and MT, Verhoff Van Gieson | Pregnant showed fewer cells but similar density of blood vessels in the lamina propria and markedly less dense tissue and collagen packing in all layers compared to virgin. Parous had the vaginal architecture similar to that of virgin sheep (epithelial height, vessel density, densely packed collagen). Parous less elastin fibers than virgin. |
|  |  |  |  | IHC for α-SMA | Pregnant had the highest proportion of muscularis; and it was significant more than virgin sheep.  No significant difference between parous and virgin. |
| Parkinson, 2016 [32] | Sheep; 1. Virgin (2y), 2. Parous (1-4 deliveries, 3-5y) | N=3-15 | Vagina | In vivo vaginal pressure | Higher compliance in the parous vagina; mainly at the cervical region |
| Knight, 2016 [61] | Sheep (2.5 - 3.5y); 1. Virgin, 2. Primiparous | N=7-8 | Vagina | POP-Q | Shorter perineal body and greater anterior and posterior vaginal wall descent in the parous relative to nulliparous |
|  |  |  |  | Passive biomechanics | Virgin had 51% stiffer and 36% stronger than parous; |
|  |  |  |  | Total collagen: Hydroxyproline Assay | No difference |
|  |  |  |  | Histology: MT, picrosirius red | No difference in collagen fiber thickness |
| Emmerson, 2017 [35] | Sheep; 1. Virgin (2y), 2. primiparous (3-4y), 3. Multiparous (4-5y) | N=6-8 | Vagina | POP-Q, Pressure sensor | Multiparous significantly more vaginal displacement at all 3 “POP-Q” points compared to nulliparous  Multiparous vagina exerted lower pressure than virgin |
|  |  |  |  | Passive biomechanics | Multiparous weaker and more compliant than virgin |
|  |  |  |  | Histology: HE, MT, Hart´s elastic  Picro-Sirius Red  IHC: α SMA | No difference in collagen organization; Multiparous has thinner muscularis, primiparous and multiparous increased elastic fibers compare to nulliparous. |
|  |  |  |  | Total collagen: Hydroxyproline Assay | Primiparous significantly less collagen compares to nulliparous |
| Rynkevic et al., 2017 [34] | Sheep; 1. Virgin (9m), 2. Pregnant (2 previous deliveries, 3y) 3. Multiparous (1y after 3rd delivery, 4y) | N=5 | Vagina | Passive Biomechanics | Pregnant sheep 64% more compliant vagina than virgin and 47% more than multiparous; Parous sheep has weaker vagina than virgin |
|  |  |  |  | Histology: Miller's Elastica | Pregnant and parous have less collagen and more elastin and smooth muscle than virgin. |
| Young et a., 2017 [33] | Sheep; 1. Virgin (2y), 2. Multiparous (1-4 delivery, 3-5y), 3. Multiparous (no birthing history, 6y) | N=14-56 | Vagina | POP-Q | Ovine vaginal displacement was seen in 50.9 % of parous  ewes and was strongly associated with parity. Nulliparous:  minimal vaginal wall displacement. |
| Hympanova et al., 2019 [36] | Sheep; 1. Neonatal (1d), 2. Prepubescence (0.3y),  3. nulliparous (1y), 4. Primiparous (1y PP, 2y), 5. Multiparous (7y), 6. menopausal (7y) | N=6 | Vagina | Passive biomechanics | Distal vagina increase compliance in primiparous, this is reversed later. |
|  |  |  |  | Active contractility | No difference |
|  |  |  |  | Vagina size | Increase in width and length in primiparous, decreasing in multiparous. |
|  |  |  |  | Histology: HE, PAS, MT, Miller’s Elastica | Increased epithelial thickness in multiparous. Decrease collagen and increased elastin after first and multiple deliveries. |
| Rynkevic et al., 2019 [37] | Sheep; 1. Virgin (9m), 2. Pregnant (3y, 2 previous deliveries), 3. Multiparous (4y, 3 deliveries) | N=5 | Vagina  cervix, uterus, bladder, rectum, and muscles (external anal sphincter and levator ani muscle) | Passive biomechanics | Vaginal wall and cervix more compliant in pregnant compared to virgin; no difference between virgin and multiparous |
|  |  |  |  | Histology: Miller | Pregnant vagina has less collagen, more elastin and more smooth muscle than virgin; Multiparous has less collagen, more elastin and more smooth muscle than virgin. |
| Urbankova et al., 2019 [27] | Sheep (1-3.5y); 1. Virgin, 2. Primiparous (1 PP), 3. Multiparous after OVX, 4. Multiparous after OVX with HRT | N=6 | Vagina | Passive Biomechanics | Primiparous: Increased compliance in the distal vagina; |
|  |  |  |  | Active contractility | Primiparous: decrease in 64% de SM contraction to KCl in the distal vagina; |
|  |  |  |  | Dimensions | Primiparous: wider and longer vagina |
|  |  |  |  | Histology: HE, PAS, MT, Miller’s pentachrome | Primiparous: 79% more elastin and 29% less collagen only in the distal vagina. |
|  |  |  |  | IHC for α-SMA and ER-α | Primiparous: Lower expression of ER-α in the epithelium of the distal vagina; tendency to decrease α-SMA expression in the distal vagina. |
| Mattsom et al., 2004 [65] | Baboon; 1. Virgin (4.8y) 2. Multiparous (23y) | N=6 | Vagina | POP-Q | Multiparous showed no evidence of POP or differences in POP-Q from nulliparous |
| Feola et al., 2010 [8] | Rhesus Macaques (9-19y); 1.Virgin, 2. parous (minimum 1y PP) | N=7 | Vagina | POP-Q | Greater descent in parous: shorter distance Hymen-cervix and Hymen-fornix |
|  |  |  |  | Passive biomechanics | Increased compliance and decrease tensile strength in parous |
|  |  |  |  | Histology: MT, Sirius red  IHC: collagen I,III,V | Loss of collagen alignment in parous but not difference in collagen subtypes |
| Kramer et al., 2006 [63] | Squirrel monkey (parous); 1. POP (13y) 2. non-POP (15y) | N=7 | PFM (LAM and obturator internus) | MRI – muscle volumes  Muscle volumes and weights from necropsy after MRI | Levator ani volumes were higher in parous with POP, obturator internus did not differ. |
| Pierce et al., 2007 [66] | Squirrel monkey, 1. Virgin (9y), 2. Parous non-POP (17y), 3. Parous POP (13y) | N=4-7 | PFM (LAM and paravaginal attachments) | Histology: HE, MT, Van Gieson | Signs of myogenic damage were found more often in the pubocaudalis muscle than the iliocaudalis, yet did not differ between POP and non- POP |
|  |  |  |  | IHC: for WGA-TRITC, My-32 and tunnel | Increased diameter of myocyte in POP; no increase in apoptosis; no disruption or atrophy of LAM |
| Bracken, 2011[9] | Squirrel Monkeys (nulliparous, 4-5y); longitudinal evaluation 1. non pregnant 2. 3d PP 3. 4m PP | N=8 | PFM (LAM, obturator internus, and COC) | MRI: volume of PFM and bladder neck and cervix position | Only COC increased volume after delivery but it recovers. Parity lead to descent of bladder neck and cervix without recovery. |
| Joyce et al., 2014 [62] | Squirrel monkey (parous); 1. POP (12y) 2. non-POP (9y) | N=19-36 | - | MRI: Correlation of POP with pelvic outlet diameter, age, parity and weight | Only parity shows a strong correlation with POP. Pelvic outlet diameter size does not contribute to POP. |
| Lindo, 2015 [64] | Squirrel Monkey (nulliparous, 3.9y); longitudinal valuation 1. VD 2. CS  Examination prior pregnancy, 1-5d after delivery, 3m after delivery | N=10 | PFM (LAM, obturator internus, and COC) | MRI: muscle volumes and bladder neck position | Volume of LAM and obturator internus did not differ between groups and decreases immediately after pregnancy; COC increase only after VD; Bladder neck descends by 3m postpartum in both groups. |

POP: pelvic organ prolapse; POP-Q: pelvic organ prolapse quantification system, PFM: pelvic floor muscle; MRI: magnetic resonance imaging; MT: Masson’s Trichrome; PAS: periodic acid shiff, IHC: immunohistochemistry; LAM: levator ani muscle; HE: Hematoxylin and Eosin; GAPDH: glyceraldehyde 3-phosphate dehydrogenase, Mmp1: metalloproteinase 1, Timp1:inhibitor of metalloproteinases 1; Col1a2: collagen I; WGA: wheat Germ Agglutinin - Myocyte; My-32: fast skeletal myosin heavy chain; ER- α: estrogen receptor alpha; COC: coccygeus muscle, PP: post-partum, CS: cesarean section, VD: vaginal delivery, PAS: periodic acid-Schiff. Y: year, d: day, w: week, h: hour; TEM: transmission electron microscopy, SVD: simulated vaginal delivery, GAGs: glycosaminoglycans, GH: genital hiatus, TVL: Total vaginal length, OVX: ovariectomy, HRT: hormonal replacement therapy; SM: smooth muscle

Supplementary table 2: Studies reporting the effect of denervation on pelvic floor muscles in relevant species. Only studies on squirrel monkeys were found.

| **Reference** | **Species and groups** | **N per group and time point** | **Tissue and Time Point** | **Outcome measures** | **Main effects** |
| --- | --- | --- | --- | --- | --- |
| Pierce et al., 2003 [41] | Squirrel Monkey (nulliparous, 2-3 y, one 14y); Neurectomy of pudendal or LAM nerve. | N=2-4 | PFM, 2 w after neurectomy | Gross anatomy | Intrapelvic anatomy is similar to humans, but pudendal nerve does not innervate PFM, as in humans |
|  |  |  |  | IHC for WGA | Only LAM neurectomy lead to muscle atrophy |
|  |  |  |  | Koele Staining (motor endplate) | One endplate zone per muscle |
|  |  |  |  | Muscle Mass and myocyte size | Only LAM neurectomy lead to loss of mass of pubocaudalis and iliocaudalis |
| Pierce et al., 2008 [67] | Squirrel Monkey (2 to 25 y); 1. Virgin non-POP (5.9y), 2. Parous non-POP (15.4y), 3. Parous POP (11.1y), 4. Bilateral neurectomy of LA (5y); | N=6-17 | LAM, 2-3y after neurectomy  PFM, 2-3y after neurectomy | MRI with or without abdominal pressure (volume of LA and position of bladder and cervix) | LAM Denervation caused a decrease in the LAM volume; It also seems to accelerate the descent of bladder after parturition (57% of the cases after 2nd delivery) |
|  |  |  |  | Gross anatomy | Intrapelvic anatomy is similar to humans, but pudendal nerve does not innervate PFM, as in humans |

POP: pelvic floor prolapse; PFM: pelvic floor muscle; MRI: magnetic resonance imaging; LAM: levator ani muscle; WGA: wheat Germ Agglutinin – Myocyte, IHC: immunohistochemistry

Supplementary table 3: Studies reporting effects of simulated vaginal birth.

| **Reference** | **Species and groups** | **N per group and time point** | **Tissue investigated and time points** | **Outcome measures** | **Main effects** |
| --- | --- | --- | --- | --- | --- |
| Damaser, 2005 [71] | Rat (age unknown); 1. Virgin, 2. Virgin SVD (VD 3ml for 1h) | N=4-5 per group and time point. | Vagina, immediately before, immediately after, 15min and 1h after SVD | Vascular perfusion by Microspheres | Decrease perfusion in the vagina at 0 min but it recovered at 5 min |
|  |  |  |  | IHC for Hypoxiprobe | Significant hypoxia of the of the stratum spinosum of the vaginal epithelium and low level of hypoxia in the muscularis |
| Woo, 2007 [70] | Rat (age unknown); 1. Virgin sham (only anesthesia), 2. Virgin controls (no interventions), 3. Virgin SVD (VD 3ml for 4h) | N=4 | Vagina, 0h and 24h after SVD | Gene expression: MCP-3 and SDF-1 | Upregulation of MCP-3 immediately and 24h after VD and downregulation of SDF1 immediately after VD. |
| Wood, 2008 [72] | Rat (10w); 1. Virgin sham, 2. Virgin SVD (VD 3ml for 1, 4 and 6h) | N=5 | Vagina, 0h after SVD | Gene expression: SDF-1, CXCR-4, CCR 1, CCR-2, CCR-3, CCR-5, VEGF, MCP-3, Il-8, HIF--1α | No difference at any time duration of VD |
| Alperin, 2010 [73] | Rat (3m); 1. Virgin, 2. Virgin SVD (VD 5ml, hanging 130g for 2h) | N=10 for histology and n=8 for passive biomechanics | Vagina, 4w after SVD | Passive biomechanics | Increase compliance, decrease tensile strength after SVD |
|  |  |  |  | Histology: MT | Disruption of fibromuscular layer |
|  |  |  |  | IHC for collagen subtypes I, III, V | Decrease in I/V collagen ratio after SVD; no difference in I/III ratio |
|  |  |  |  | Size of vagina (genital hiatus (GH) and total vaginal length (TVL)) | 1.2-1.5-fold increase in diameter |
| Ruano, 2011 [53] | Rat; 1. Virgin, 2. Virgin SVD, 3. CS, 4. CS + SVD, 5. Spontaneous delivery, 6. Late pregnancy (d20); SVD: VD 5ml, hanging 100g for 3h | N=10 | Vagina, 4d or 3m after delivery or SVD | GAG biochemical analysis | Pregnancy and spontaneous delivery decrease the levels of GAGs at 4d; spontaneous delivery or SVD increase GAGs at 3m |
| Downing, 2013 [69] | Rat (age unknown); 1. Virgin, 2. Virgin SVD 3. Spontaneous delivery, 4. CS; SVD: VD 2.5ml, hanging catheter for 3h; still inflated catheter when removed | N=5-6 | Vagina, 2d and 2w after delivery or SVD | Gene expression (genes involved in elastic fiber homeostasis): Loxl-1, Fbln5, tropoelastin | Vaginal delivery, SVD and pregnancy cause upregulation of genes involved in elastic fiber homeostasis. |
| Downing, 2013 [58] | Rats; 1. Virgin (12-15w), 2. Virgin SVD, 3.Spontaneous delivery, 4. CS, 5. Multiparous (9-12m); SVD: VD 2.5ml, hanging catheter for 3h; still inflated catheter when removed | N=6 | Vagina, 2d and 2w after delivery or SVD | Passive Biomechanics (Pressure-infusion system) | SVD at 2d and Multiparous vagina have a higher compliance than virgin; it normalizes at 2w. |
|  |  |  |  | Histology: Van Giesen – Tortuosity Hart s - Morphometric analysis of elastin fibers | 2 days after delivery, SVD and in Multiparous significantly higher tortuosity of elastic fibers compare to virgin; at 2w virgin, SVD, spontaneous delivery and CS showed similar elastic fibers |
| Catanzarite, 2018 [74] | Rats (3m); 1. Virgin control, 2. Virgin SVD, 3. Pregnant-late control (20-21d), 4. Pregnant SVD, 5. Intrapartum (spontaneous delivery); SVD: VD 1-5ml, hanging 130g for 1-2h | N=10-22 | PFM, 0h after SVD | Fiber and sarcomere length of PFM | SVD causes acute and progressive stretch of the myofibers at higher distention volumes, mainly coccygeus and pubocaudalis. At 3ml mimics the spontaneous vaginal delivery. SVD in virgin causes a significant longer sarcomere length compared to pregnancy |
|  |  |  |  | Transmission electron microscopy (TEM) | SVD causes distortion of Z-lines and misalignment of adjacent sarcomeres mainly in Coccygeus and pubocaudalis |
|  |  |  |  | Genital hiatus, total vaginal length, and perineal body | Genital hiatus increased during vaginal distention in both nonpregnant and pregnant |
| Callewaert, 2020 [68] | Rats (12 to 15 w); 1. Virgin control, 2. Virgin SVD; SVD: PNC + VD 3ml for 4h | N=6 except for PCR (n=5) | Vagina, 3d and 1, 2, 3 and 6w after SVD | IHC for CD34, α-SMA, PPG9.5 | SVD cause rupture of smooth muscle layer with scar deposition; Loss of microvasculature at 1w and 6w; no difference in PPG9.5 expression |
|  |  |  |  | Active contractility | Hypersensitivity to carbachol at 2 and 3w after SVD; Hyposensitivity at 6w |
|  |  |  |  | Gene expression: caldesmon, muscarinic receptor 2 and 3, smoothtelin, rock 1, col1a1, Col3a2, | At 3d: upregulation of smoothelin, rock 1 and muscarinic receptor 2; at 1w: downregulation of caldesmon and upregulation of collagen III |

SVD: simulated vaginal birth; VD: Vaginal distention; PNC: pudendal nerve crush; α-SMA: smooth muscle actin; MT: Masson`s Trichrome; PFM: pelvic floor muscle; MCP-3: Monocyte chemotactic protein-3; SDF-1: Stromal derived factor-1; VEGF: vascular endothelial growth factor; FBLN5: Fibulin 5; CS: cesarean section h: hour, IHC: immunohistochemistry, min.: minute

Supplementary table 4: Studies reporting the effect of iatrogenic menopause in relevant species

| **Reference** | **Sp. And Groups** | **N per group and time point** | **Tissue investigated and time points** | **Outcome measures** | **Main effects** |
| --- | --- | --- | --- | --- | --- |
| Önol et al., 2006 [82] | Rats (6m); 1. Intact mature, 2 OVX mature. | N=15 | Vagina, 6w after OVX | Active contractility | OVX decrease contractility only of distal vagina to EFS and decreased response to phenylephrine and α -1 and 2 blockade; no difference to carbachol. No effect on the proximal vagina |
|  |  |  |  | Histology | OVX caused smooth muscle and epithelium layer atrophy. |
|  |  |  |  | TEM | OVX induced severe degeneration of epithelial layer |
| Rizk et al, 2007 [79] | Rats; 1. Intact young (3m), 2. OVX young (3m), 3. Intact old (18m), 4. OVX old (18m) | N=6 | LAM, 4w after OVX | WB: Isomyosin and p27^kip1^ | OVX increased isomyosin in old rats and p27^kip1^ in both young and old rats |
| Rizk et al, 2007 [83] | Rats; 1. Intact old (13m), 2. OVX old, 3.OVX+E2, 4. OVX ghrelin, 5. OVX E2+ghrelin; treatment started 4w after OVX | N=6 | LAM, 10w after OVX | WB: p27^kip1^ | OVX increased 44% of p27^kip1^; estrogen restored the normal levels and ghrelin decreases to below sham levels |
| Moalli et al., 2008 [78] | Rats; 1. virgin young (4m), 2. Parous Young (4m), 3. Parous middle age (9m); all with subgroups: a. intact, b. OVX, c.OVX+CMT8, d. OVX+E2, e. OVX+E2+P4 | N=34-40 | Vagina, 8w after OVX | Passive biomechanics | Increased compliance and decrease ultimate load after OVX, only in young rats; CMT8, E2 and E2+P4 restored tissue quality. |
| Zong et al., 2009 [84] | Rats (3m); 1. Intact, 2. OVX, 3. OVX E2+P4 | N=6-9 | Vagina, 8w after OVX | WB: Mmp13 | OVX increase activity of Mmp13; this was suppressed by E2 + P4 supplementation |
| Basha et al., 2013 [80] | Rat (3-4m); 1. Intact, 2.OVX, 3. OVX+E2 (Starting 2w after OVX) | N =3 for histology; n=5 for active contractility and protein analysis; n=6 for estradiol | Vagina, 3w after OVX | Active contractility of the proximal vagina | OVX decrease contractility to KCL, E2 reverse the effect |
|  |  |  |  | Serum Estradiol | OVX decrease estradiol levels |
|  |  |  |  | Gene expression: MHC isoforms, carboxyl terminal myosin heavy chain and caldesmon | OVX downregulate %SM1 in MHC, E2 reverse the effect. |
|  |  |  |  | WB: caldesmon and MHC | Decrease in caldesmon (2x); E2 reverse the effect; no effect in MHC |
|  |  |  |  | Coomasi staining of carboxyl terminal isoforms | Decrease in SM1, not SM2, E2 reverse the effect. |
|  |  |  |  | Histology (MT) | OVX decrease epithelial thickness and leads to thinning of the vaginal wall. No difference in the muscularis. E2 reverse the effect |
| Liang et al., 2016 [75] | Rat (4m); 1. Intact, 2. OVX, 3. OVX+E2 | N=8 | Vagina, 8w after OVX | Passive Biomechanics | OVX cause increase compliance (34%) and decrease tensile strength (16%). E2 restored to intact levels. |
|  |  |  |  | Biochemistry (total collagen and GAG) | No difference between the groups |
| Mao et al., 2019 [77] | Rat (8w); 1. Intact young, 2. OVX | N=6 | Vagina; 2, 4 and 16w after OVX | Passive biomechanics | OVX caused decreased compliance and decreased ultimate load at 16w |
|  |  |  |  | WB: Col1 and Col3 | OVX increased col1 and decreased col3 at 2w |
|  |  |  |  | IHC for α-SMA | OVX decrease the fraction of α-SMA and smooth muscle bundles become disorganized at 2w |
|  |  |  |  | Histology (HE and sirius red) | Atrophy of epithelium at 16w; Decrease in immature collagen and increase in mature collagen at 16w |
| Kim et al., 2004 [76] | Rabbit (age unknown); 1. Intact, 2. OVX, 3. OVX+E2, 4.OVX + testosterone | N=4-7 | Vagina, 4w after OVX | Active contractility | No difference |
|  |  |  |  | Histology | OVX: thinning of vaginal wall, atrophy of vaginal epithelium and muscularis; E2 restore epithelium but only partially the muscularis; Testosterone restore muscularis but only partially epithelium. |
| Lemmex et al., 2016 [81] | Rabbit; 1. intact adult (1y), 2. OVX adult (1y14w) 3. OVX adolescent (48w) | N=7-8 | Medial collateral ligaments; 14w after OVX (adult) and 33w after OVX (adolescent) | Passive Biomechanics | OVX leads to higher compliance in adults but not adolescents, which showed only higher strain |
|  |  |  |  | Biochemistry (collagen and GAG) | Only adult showed higher total collagen |
|  |  |  |  | Gene expression: Biglycan, Col-I, Col-III, Col-V, decorin, estrogen receptor, Mmp1, Mmp3, Mmp13, Progesterone receptor, Lubricin, Timp1, Timp2 Timp3 | Upregulation of lubricin and col1 in adults; downregulation of progesterone receptor in adolescents |
| Hympanova, et al. 2019 [36] | Sheep (7y); 1. Intact Multiparous 2. OVX Multiparous | N=6 | Vagina, 160d after OVX | Passive biomechanics | No difference |
|  |  |  |  | Active contractility | No difference in the distal vagina; Middle vagina lower contractility to KCl |
|  |  |  |  | Vagina size | OVX shorter and narrower vagina |
|  |  |  |  | Histology | OVX higher collagen and lower elastin; OVX sheep had less glycogen and epithelium atrophy in both distal and middle vagina |

OVX: ovariectomy; EFS: electro field stimulation; GAG: glycosaminoglicans: MHC: Myosin Heavy Chain; α-SMA: smooth muscle actin; E2: estrogen (estradiol); P4: progesterone; CMT-8: chemically modified tetracycline-8 (matrix metalloproteinase inhibitor), WB: western blot, m: month, w: week, y: year; LAM: levator ani muscle; Mmp13: matrix metalloproteinase 13, MT: Masson`s Trichrome, HE: Hematoxylin & eosin, Col: collagen

Supplementary table 5. Studies reporting the effect of aging in relevant species

| **Reference** | **Species and groups** | **N per group and time point** | **Tissue investigated and time points** | **Outcome measures** | **Main effects** |
| --- | --- | --- | --- | --- | --- |
| Jiang et al., 2014 [85] | \| Mouse; 1. Young (3m), 2. Young accelerated aging model (3m; Busulfan), 3. Old (18m) \| \| --- \| \| \| | N=20, except for blood samples (n=3-4) | Vagina, 1 m after Busulfan (accelerated aging model) | Serum Estradiol | Accelerated aging leads to reduction in 62% of estradiol while aging led to 44% reduction |
|  |  |  |  | Gene expression: elastin, Lox, (Lox 1, 2, 3, 4) | Downregulation of all LOX family and elastin in both aging and accelerated aging |
|  |  |  |  | WB: Lox 3, Lox 4 | Downregulation of LOX3, Lox4 in both aging and accelerated aging |
| Rizk et al, 2007 [79] | Rats; 1. Intact young (3m), 2. OVX young (3m), 3. Intact old (18m), 4. OVX old (18m) | N=6 | LAM, 4w after OVX | WB: Isomyosin and p27kip1 | Aging increased both isomyosin and p27kip1 in old rats |
| Moalli et al., 2008 [78] | Rats; 1. virgin young (4m), 2. Parous Young (4m), 3. Parous middle age (9m); all with subgroups: a. intact, b. OVX, c.OVX+CMT8, d. OVX+E2, e. OVX+E2+P4 | N=34-40 | Vagina | Passive Biomechanics | No difference related to aging, only OVX |
| Shveirky et al., 2019 [86] | Rats; 1. Young (3m), 2. Old (12m) | N=5 | Vagina | Passive Biomechanics 30d | No difference in the strength of young and old rats without injury at higher compliance in old rats; after injury young animals regained about 60% of the strength and compliance whereas old rats regain only 15% of its original force and modulus. |
|  |  |  |  | Wound healing 1, 3, 7, 14d post injury | At 3d wound is completely closed at in young rats; old rats have granulation tissue at 7d, no complete healing. |
|  |  |  |  | Gene expression: MIF at 30d | Old rat had delayed and prolonged expression of MIF. |
|  |  |  |  | Estradiol levels 0d | Old rats showed lower levels of E2 |
| Mattsom et al., 2005 [65] | Baboon; 1. Virgin young (5y), 2. Aged multiparous (23y) | N=6 | Vagina | POP-Q | No clinical signs of POP |

WB: western blot, d: day; m: month, y: year; OVX: ovariectomy, LOXL 1-4: Lysyl oxidase-like proteins 1-4, LAM: levator ani muscle; CMT-8: chemically modified tetracycline-8 (matrix metalloproteinase inhibitor); MIF: macrophage-migration inhibitory factor; POP: pelvic organ prolapse; POP-Q: pelvic organ prolapse quantification system; E2: estradiol, P4: progesterone;

Appendix 1.

**ANIMAL MODEL FOR POP – SEARCH STRATEGY:**

**Note:** search done 6.9.2019;

PUBMED:

Advanced, combine the 2 concepts with NOT (so delete the first NOT of concept 1)

EMBASE:

Advanced, switch off all embase mapping options, search 1^st^ concept (delete the NOT in the beginning), search the second concept and then write #2 NOT #1

WOS:

Advanced, search just the core Collection.

**Concept 1: animal model**

### **PUBMED**

NOT (human[Mesh] NOT animal[Mesh:NoExp])

**EMBASE**

NOT (‘human’/exp NOT ('animal'/de OR 'coelenterate'/exp OR 'Mesozoa'/exp OR 'Placozoa'/exp OR 'sponge (Porifera)'/exp OR 'Deuterostomia'/de OR 'Ambulacraria'/exp OR 'Bilateria'/de OR 'Coelomata'/exp OR 'Protostomia'/exp OR 'Pseudocoelomata'/exp OR 'Chordata'/de OR 'Cephalochordata'/exp OR 'Hyperotreti'/exp OR 'Urochordata'/exp OR 'mammal'/de OR 'calf (mammal)'/exp OR 'monotreme'/exp OR 'amniote'/de OR 'reptile'/exp OR 'sauropsid'/exp OR 'tetrapod'/de OR 'Amphibia'/exp OR 'vertebrate'/de OR 'fish'/exp OR 'therian'/de OR 'marsupial'/exp OR 'placental mammal'/de OR 'Afrotheria'/exp OR 'Boreoeutheria'/exp OR 'Laurasiatheria'/exp OR 'Xenarthra'/exp OR 'Euarchontoglires'/de OR 'Dermoptera'/exp OR 'Glires'/exp OR 'Scandentia'/exp OR 'Haplorhini'/de OR 'tarsiiform'/exp OR 'primate'/de OR 'prosimian'/exp OR 'simian'/de OR 'Platyrrhini'/exp OR 'Catarrhini'/de OR 'Cercopithecidae'/exp OR 'ape'/de OR 'Hylobatidae'/exp OR 'hominid'/de OR 'orangutan'/exp OR 'Homo neanderthalensis'/exp OR 'gorilla'/exp OR 'chimpanzee'/exp OR 'nonhuman'/exp OR 'animal experiment'/exp OR 'model'/exp))

**WOS**

animal OR animal model OR mouse* OR mice* OR rodent* OR rat OR rats OR bovine OR rabbit* OR sheep OR ewe* OR primate* OR monkey* OR goat* OR hamster* OR swine* OR pig* OR porcine OR dog* OR beagle*

**Concept 2: Pelvic organ prolapse**

### **PUBMED**

"Pelvic Organ Prolapse"[Mesh] OR pelvic-organ-prolapse*[tiab] OR urogenital-prolapse*[tiab] OR vaginal-vault-prolapse*[tiab] OR cystocele[tiab] OR cystocoele[tiab] OR "urinary bladder prolapse"[tiab] OR rectal-prolapse*[tiab] OR anus-prolapse*[tiab] OR uterine-prolapse*[tiab] OR vaginal-prolapse*[tiab] OR "genital prolapse"[tiab] OR "genito-urinary prolapse"[tiab] OR "genitourinary prolapse"[tiab] OR "pelvic descent"[tiab] OR "pelvic organ descent"[tiab] OR "pelvic prolapse"[tiab] OR "vaginal descensus"[tiab] OR "vaginal descent"[tiab] OR "vaginal wall prolapse"[tiab]

**EMBASE**

'pelvic organ prolapse'/exp OR 'pelvic organ prolapse*':ti,ab,kw OR 'urogenital prolapse*':ti,ab,kw OR 'vaginal vault prolapse*':ti,ab,kw OR cystocele:ti,ab,kw OR cystocoele:ti,ab,kw OR 'urinary bladder prolapse':ti,ab,kw OR 'rectal prolapse*':ti,ab,kw OR 'anus prolapse*':ti,ab,kw OR 'uterine prolapse*':ti,ab,kw OR 'vaginal prolapse*':ti,ab,kw OR 'genital prolapse*':ti,ab,kw OR 'genito urinary prolapse*':ti,ab,kw OR 'genitourinary prolapse*':ti,ab,kw OR 'pelvic descent':ti,ab,kw OR 'pelvic organ descent':ti,ab,kw OR 'pelvic prolapse*':ti,ab,kw OR 'vaginal descensus':ti,ab,kw OR 'vaginal descent':ti,ab,kw OR 'vaginal wall prolapse':ti,ab,kw

**WOS**

“pelvic organ prolapse” OR “urogenital prolapse” OR “vaginal vault prolapse*” OR cystocele OR cystocoele OR “urinary bladder prolapse” OR “rectal prolapse” OR “anus prolapse*” OR “uterine prolapse*” OR “vaginal prolapse*” OR “genital prolapse” OR “genito-urinary prolapse” OR “genitourinary prolapse” OR “pelvic descent” OR “pelvic organ descent” OR “pelvic prolapse” OR “vaginal descensus” OR “vaginal descent” OR “vaginal wall prolapse”

**Appendix 2:**
